# Single-Site Endoscopic Surgery for Soft Tissue Lesions: An Innovative Technique

**DOI:** 10.7759/cureus.76386

**Published:** 2024-12-25

**Authors:** Chong Xie, Zhengtuan Guo, Weilong Lin, Peihua Wang, Weijia Yang, Huaijie Wang

**Affiliations:** 1 Department of Pediatric Surgery and Vascular Anomalies, Xi’an International Medical Center Hospital, Xi'an, CHN

**Keywords:** capillary lymphatic venous malformation, endoscopy, fibroadipose vascular anomaly, kaposiform hemangioendothelioma, klippel-trénaunay syndrome, lymphatic malformation, lymphedema, surgery, venous malformation

## Abstract

Purpose

We aimed to report an innovative single-site endoscopic surgery for soft tissue lesions performed at our center.

Methods

All patients who underwent soft tissue surgery were reviewed. All consecutive patients who underwent single-site endoscopic surgery between September 2019 and March 2024 were included in the study. Data were extracted from our medical records database, including sex, age, diagnosis, sites of surgery, surgery, operation time, blood loss, and follow-up.

Results

There were 10 females and five males in the current study, with ages ranging from one year to 26 years (median = nine years). Conditions for surgery included enlargement of the leg (n = 5), fibro-adipose vascular anomaly (n = 4), microcystic lymphatic malformation (n = 3), venous malformation with thrombosis (n = 3), and borderline tumor (n = 2). Surgical sites included the lower extremity (n = 13) and upper extremity (n = 2). Perioperative thrombosis prophylaxis included elastic compression and subcutaneous low-molecular-weight heparin in patients who had venous malformation and localized intravascular coagulopathy for three or more days. Surgery included tumor en bloc resection, tumor partial resection, lymphedema debulking, microcystic lymphatic malformation debulking, limb debulking, intramuscular lesion radical resection, thrombectomy, Achilles lengthening, relaxation of the ankle capsule, gastrocnemius recession, and tendon transfer. Technical success was obtained in all patients. Operative duration ranged from 66 to 455 minutes (median = 183 minutes). Blood loss ranged from 5 to 700 mL (median = 50 mL). One patient received a blood transfusion.

Conclusions

This retrospective review demonstrates the feasibility of using a single-site endoscopic approach to resection of subcutaneous, muscular and tendinous lesions and proof of principle for future soft tissue surgery.

## Introduction

Generally, soft tissue lesions refer to dermal, subcutaneous, fascial, and muscular lesions. Open surgery is most often used for these lesions. Recently, endoscopic surgery has been introduced into the management of various soft tissue diseases, such as thyroidectomy [1], subcutaneous lipoma and tumor-like lesions [2], vascular malformation [3], and superficial venous thrombosis [3]. The soft tissue space of the human body includes subcutaneous and intermuscular space, which provides a vast operating field for endoscopic surgeons [4]. We have used two-site or multi-site endoscopic techniques to resect various vascular malformations and other benign soft tissue lesions using laparoscopic devices since 2016 [5], including microcystic lymphatic malformation (LM), Klippel-Trénaunay syndrome (KTS), venous malformation (VM), and fibro-adipose vascular anomaly (FAVA) [3,6]. More recently, we have performed single-site endoscopic surgery for subcutaneous and intramuscular lesions. Herein, we describe the application of single-site endoscopic surgery in the management of soft tissue lesions at our center. This approach has the potential to achieve better cosmetic outcomes and reduce incision-related complications. We believe this approach can broaden the spectrum of endoscopic surgery.

## Materials and methods

This study is a retrospective review of a single center. The study was approved by the Institutional Ethics Review Board of Xi’an International Medical Center. Patients who underwent soft tissue surgery from September 2019 to March 2024 were reviewed. Written informed consent about this new approach and alternative techniques was obtained from all parents or guardians. All consecutive patients who underwent single-site endoscopic surgery were included in the study. Data were extracted from our medical records database, including sex, age, diagnosis, sites of surgery, surgery, operation time, blood loss, and follow-up.

Diagnosis and treatment

Diagnosis and treatment decisions were made by our multidisciplinary team comprising pediatric surgeons, ultrasonologists, and pediatric interventional radiologists. If an unsatisfied response was predicted or non-operative therapy failed, then surgery would be recommended. Endoscopic surgery was not indicated in patients who had excessive skin requiring removal or patients with suspicious malignant lesions. Indications and (relative) contraindications of endoscopic surgery at our center were discussed and decided preoperatively. If the lesion was too difficult to expose under endoscopy due to deep location and/or infiltrating into vital structures, totally multi-site endoscopic surgery was also not recommended at our center. Single-site endoscopic surgery or endoscopy-assisted small incision surgery may be considered alternatively.

In patients with localized lesions, surgery is usually radical resection. If the lesion was extensive or infiltrative of vital structures, such as extensive microcystic lymphatic malformation, unresectable FAVA, PTEN hamartoma of soft tissue, or primary lymphedema, the goal of surgery was symptom relief and/or appearance improvement. Surgery was typically not curative and may be used for biopsy or debulking instead. Postoperatively, complementary radiological interventional therapy with ethanol, foam sclerosant, bleomycin, and/or gene sequencing-based targeted therapy with oral sirolimus, alpelisib, and trametinib may be required in some patients with vascular anomalies.

Single-site endoscopic surgery technique

**Figure 1:**
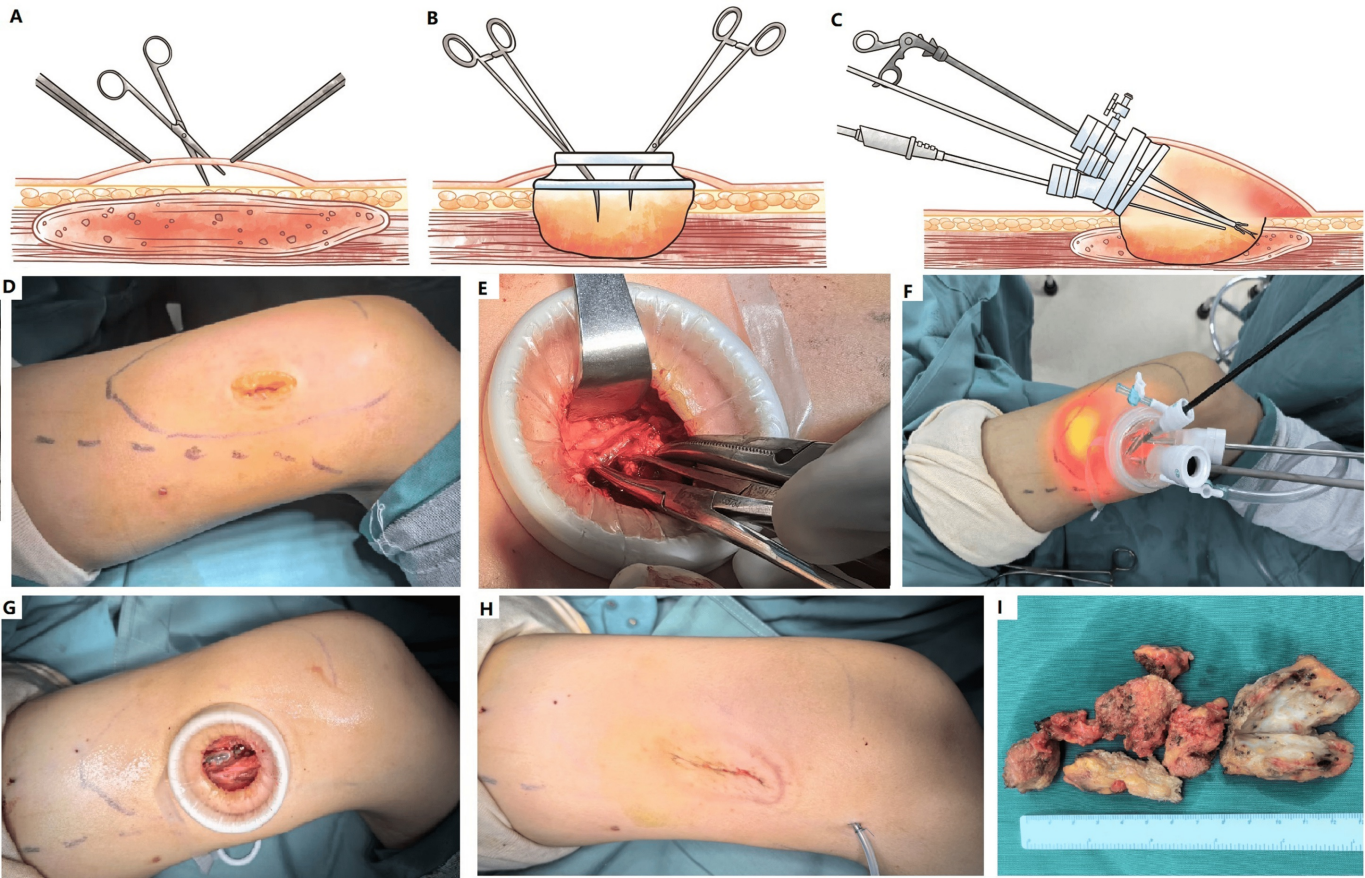
A step-by-step description of single-site endoscopic surgery. (A-C) A step-by-step description of single-site endoscopic surgery. (D) A 10-year-old boy presented with a painful mass of medial vastus muscle with Hunter's canal involvement and knee stiffness. He was diagnosed with fibro-adipose vascular anomaly (FAVA) and underwent endoscopic surgery. Initially, a 2-2.5 cm incision was made at the overlying skin of Hunter's canal, and (E) the canal and medial vastus muscle were dissected to protect femoral vessels in the canal with a fashion of open surgery via this incision. After partial resection of the lesion, the soft tissue space was created. (F) Then, a quadruple-channel port was placed to establish a single-site channel to resect the rest of the lesion. (G) The specimen was retrieved via the port. (H) The portal incision was closed, and a suction drainage tube was placed separately. (I) The typical gross appearance of intramuscular FAVA specimen. A histopathologic study confirmed the diagnosis.

**Figure 2:**
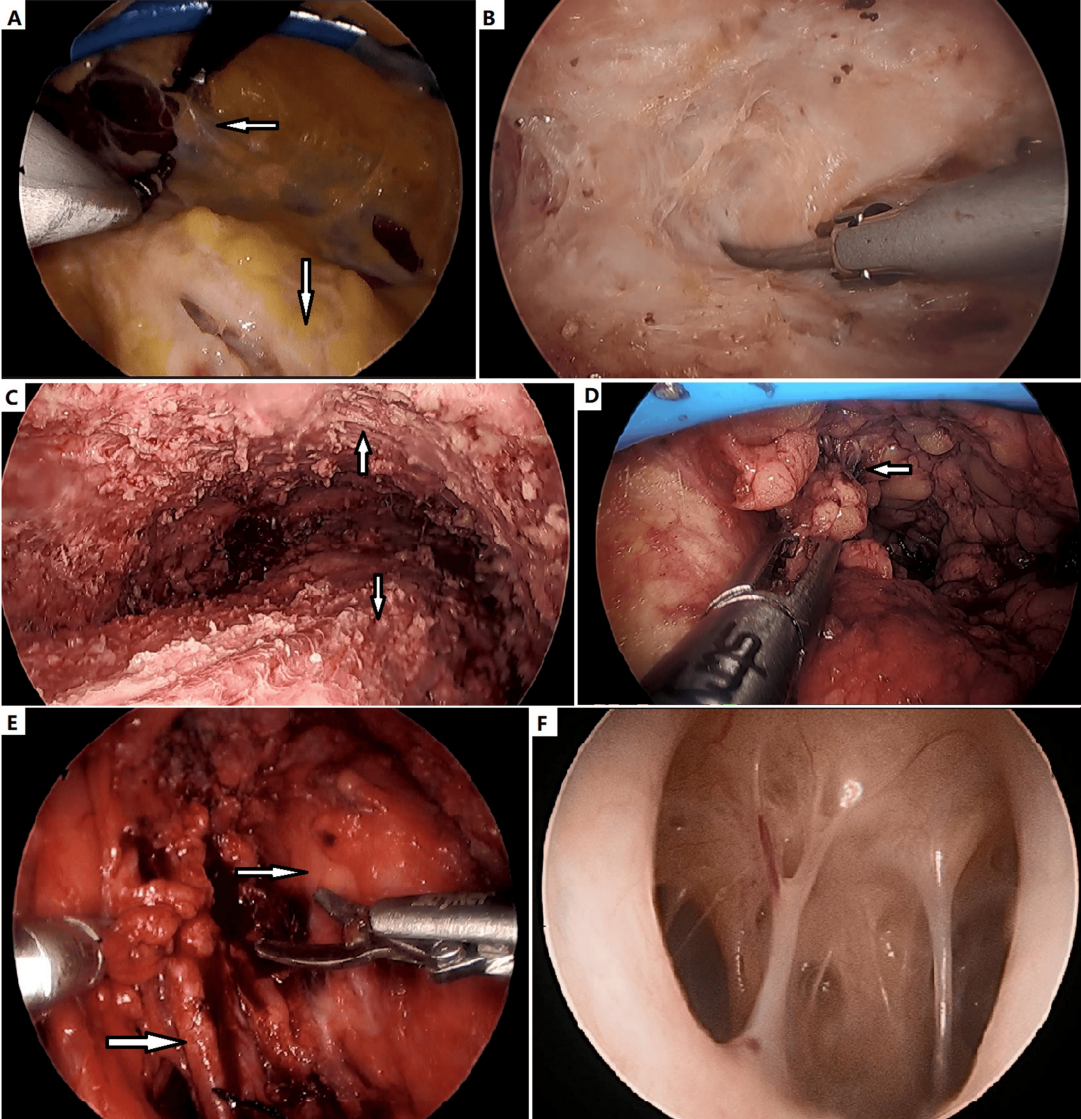
Endoscopic view of various soft tissue conditions. (A) Dividing the subcutaneous neurovascular bundles (leftward arrow) between dermis and deep fascia (downward arrow). (B) Debulking of subcutaneous microcystic lymphatic malformation (LM). (C) Muscle (downward arrow) and overlying flap (upward arrow) after resection of extensive microcystic LM and deep fascia. The flap was elevated one-third to half the circumference of the limb. (D) Resection of a superficial venous thrombosis (SVT, leftward arrow). (E) Dividing the fibro-adipose vascular anomaly (FAVA) lesion and femoral vessels (rightward arrows). (F) Inner view of cystic LM. The so-called septum of cystic LM was actually subcutaneous neurovascular bundles.

According to preoperative magnetic resonance imaging and ultrasonographic studies, skin markers were made to delineate the extent of lesions. Surgery was performed under general anesthesia with endotracheal intubation. The patient was properly positioned to optimize access to the treatment area or lesions and aid exposure during the operation. A tourniquet was used to secure pulmonary gas embolism and bleeding during the operation. Single-site endoscopic technique was different from soft tissue endoscopic surgery described previously [3]. Initially, a 2-2.5 cm incision was made at the lesion area, and a partial excision was performed in the fashion of open surgery via this incision. In this step, the lesion was resected as much as possible via this small incision. Then, a space beneath and around this incision was created. A quadruple channel port (Surgaid Starport, Xiamen, China) was placed to establish a single-site channel to resect the rest of the lesion. Carbon dioxide was insufflated at a low flow rate to a pressure of 6-12 mm Hg through the port. A 5-mm 30° laparoscope and 5-mm laparoscopic instruments (Stryker Endoscopy, San Jose, California) were then placed through the port. Subcutaneous non-vital neurovascular bundles could be divided to expose the lesion using a monopolar hook endocautery or the ultrasonic scalpel (HocerMed, Tianjin, China) (Figures 1, 2). Silk sutures may be used for lesion or muscle traction to aid the exposure if required. Intraoperative repeated rinsing with saline can be performed to increase visibility.

In patients with subcutaneous vascular malformation, the thickened deep fascia was also resected (Figure 2). When debulking lymphedema or extensive lesions, the skin flap could be elevated one-third to half the circumference of the limb. If the lesion involves more than half the circumference of the extremity, staged resection may be required to secure the flap survival. Skin flap was full thickness, but as thin as possible (Figure 2). The goal of surgery was to improve the mass effect and improve appearance. Radical resection was usually impossible.

In patients with intramuscular lesions, such as FAVA and PTEN (type) hamartoma of soft tissue, thickened deep fascia and affected muscles must be radically resected if possible, meanwhile, vital nerve and blood vessels should be protected and preserved (Figure 2). Following lesion resection, endoscopic transverse gastrocnemius aponeurotic recession can be performed if Achilles lengthening is required. Achilles tenotomy, relaxation of ankle capsule, neurolysis/neurectomy, and tendon transfer can also be completed with endoscopic technique.

After specimen retrieval and the surgical field rinsing, a suction drain was placed through a separate incision (Figure 1). After surgery, compressive elastic wrap was routinely prescribed to prevent the collection of lymphatic fluid or blood beneath the flap, and circumferential compression adhered the flap to the underlying tissue. The suction drain was also used as the access of postoperative sclerotherapy if lymph drainage persisted following LM debulking.

Stretching exercises were routinely performed to improve the range of motion of joints and promote postoperative rehabilitation in patients who underwent resection of muscular and/or tendinous lesions.

## Results

Clinical characteristics

**Figure 3:**
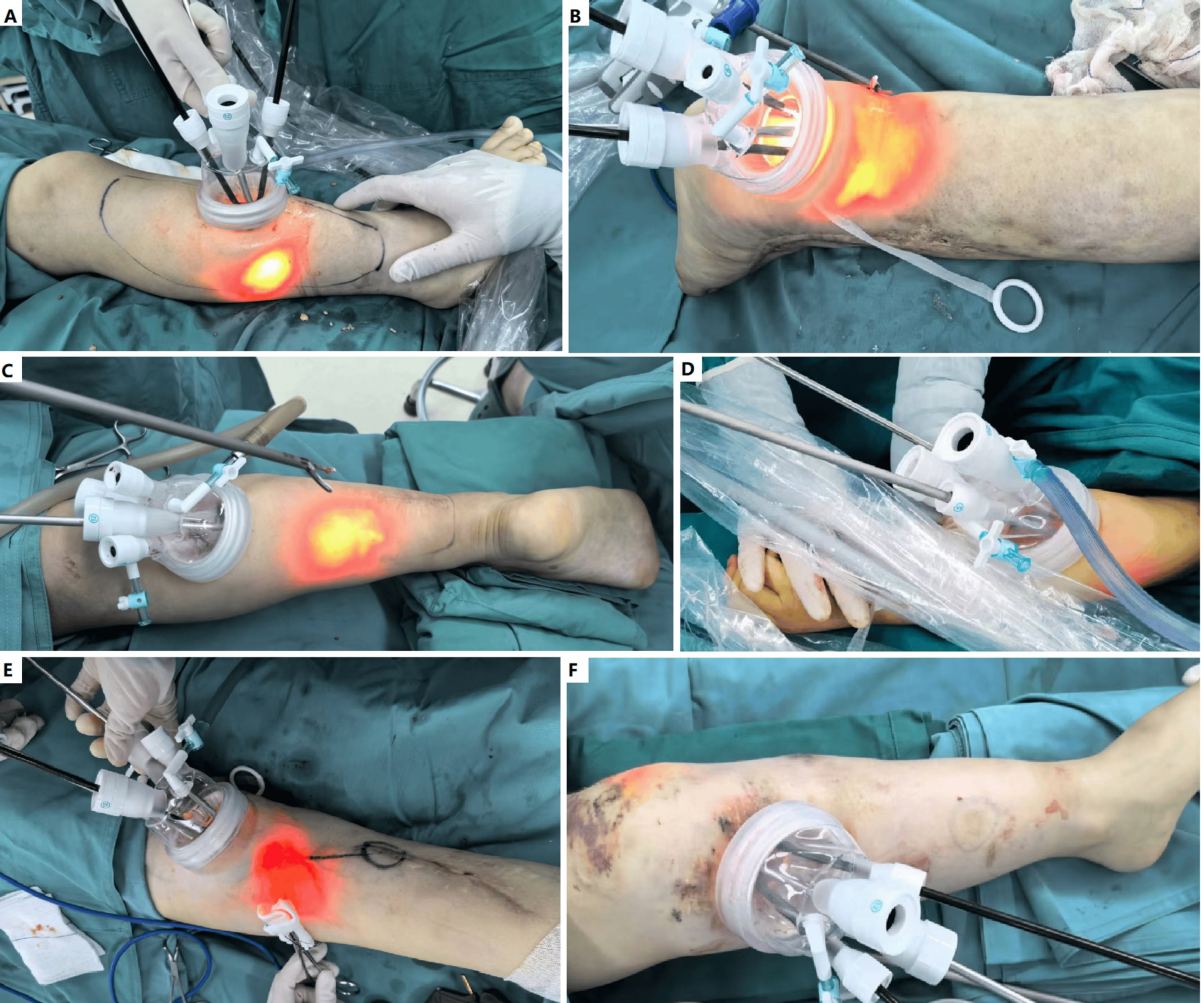
Single-site endoscopic surgery for various soft tissue conditions. (A) Primary lymphedema and dermatofibrosarcoma protuberans. The tumor was resected en bloc via the incision, then, single-site endoscopic surgery was performed to debulk the lymphedema. (B) Debulking microcystic lymphatic malformation (LM) of the leg. (C) Resection of extensive superficial venous thrombosis (SVT) from venous malformation of the leg. (D) Resection of a fibro-adipose vascular anomaly (FAVA) of the forearm. (E) Recurrent SVT and muscular venous malformation with fibrosis and contracture. (F) Resection of extensive SVT in Klippel-Trénaunay syndrome (KTS).

**Figure 4:**
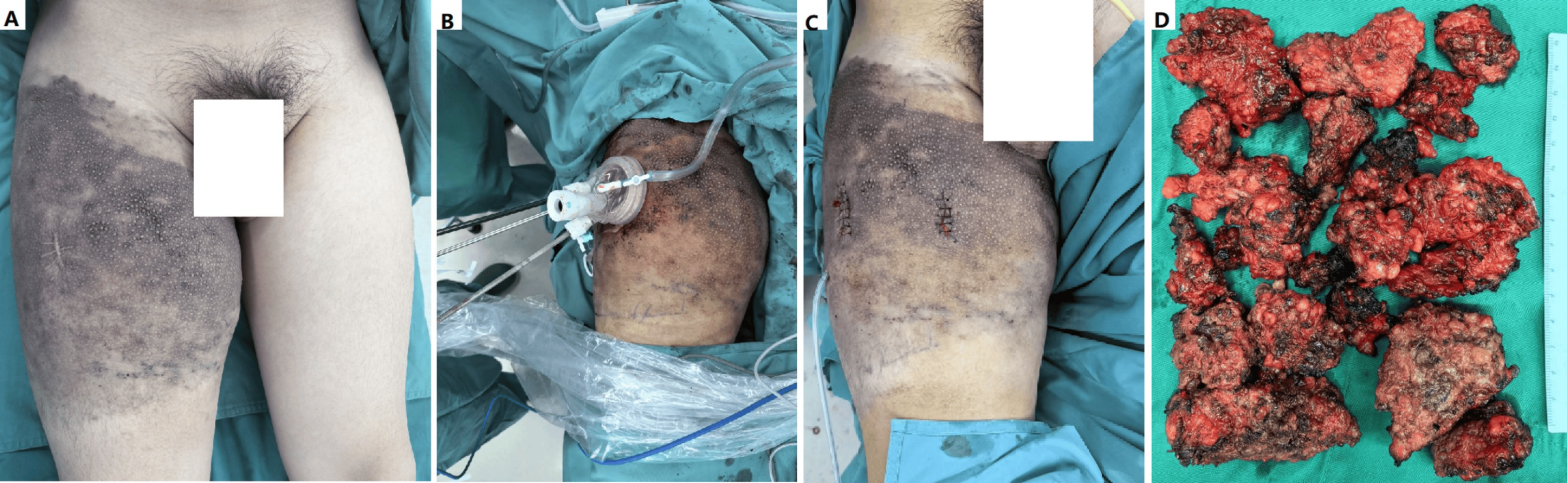
Single-site endoscopic surgery for a bulky kaposiform hemangioendothelioma. (A) A 13-year-old boy presented with a large mass in his thigh. The diagnosis of kaposiform hemangioendothelioma was confirmed by biopsy during infancy. (B and C) He underwent a single-site endoscopic partial excision. (D) The tumor was significantly debulked. A histopathologic study re-confirmed the diagnosis. He received oral sirolimus after surgery for ongoing therapy.

Soft tissue lesions in children mostly presented as subcutaneous or intramuscular, localized to bulky lesions, limb enlargements, and pain from superficial venous thrombosis, while patients with FAVA presented with an enlarging, intramuscular painful mass, local swelling, and progressively limited range of motion. There were 10 females and five males in the current study, with ages ranging from one year to 26 years (median = nine years). Conditions for surgery included enlargement of the leg (n = 5), FAVA (n = 4), microcystic LM (n = 3), VM with thrombosis (n = 3), and borderline tumor (n = 2). Surgical sites included the lower extremity (n = 13) and upper extremity (n = 2) (Table 1 and Figures 3, 4). Perioperative thrombosis prophylaxis included elastic compression and subcutaneous low-molecular-weight heparin in patients who had VM and localized intravascular coagulopathy for three or more days. Surgery included tumor en bloc resection, tumor partial resection, lymphedema debulking, microcystic LM debulking, limb debulking, intramuscular lesion radical resection, thrombectomy, Achilles lengthening, relaxation of the ankle capsule, gastrocnemius recession, and tendon transfer. Postoperative sclerotherapy with bleomycin was performed via a drainage tube in two patients with microcystic LM and kaposiform hemangioendothelioma, respectively.

**Table 1:** Clinical characteristics of the patients. KTS: Klippel-Trénaunay syndrome; LM: lymphatic malformation; FAVA: fibro-adipose vascular anomaly. Sclerotherapy*: Postoperative sclerotherapy with bleomycin via drainage tube.

No.	Gender	Age at surgery (years)	Symptoms	Sites of surgery	Diagnosis	Interventions before surgery	Surgery	Operative duration (min)	Estimated blood loss (mL)	Additional management after surgery
1	F	9	Enlargement of the leg; localized mass	Right leg	Primary lymphedema and dermatofibrosarcoma protuberans	Bleomycin sclerotherapy	Tumor en bloc resection and lymphedema debulking	213	20	Elastic compression
2	F	1	Enlargement of the leg	Right leg	KTS	Bleomycin sclerotherapy	Debulking	99	5	Elastic compression; sclerotherapy*
3	F	2	Enlargement of the leg	Left leg	Microcystic LM	Ethanol and bleomycin sclerotherapy	Debulking	141	20	Elastic compression
4	F	13	Local pain and Achilles contracture	Right leg	KTS	Ethanol and bleomycin sclerotherapy	Thrombectomy; Achilles lengthening; relaxation of the ankle capsule	110	50	Elastic compression; stretching exercises
5	F	5	Extensive superficial venous thrombosis; enlargement of the leg	Left thigh and leg	KTS	Ethanol and bleomycin sclerotherapy	Thrombectomy and debulking	183	50	Elastic compression
6	M	2	Extensive superficial venous thrombosis; enlargement of the leg	Right thigh and leg	KTS	None	Thrombectomy and debulking	88	50	Elastic compression
7	M	13	Bulky mass	Right thigh	Kaposiform hemangioendothelioma	Biopsy and oral sirolimus	Partial resection of the tumor	455	700	Oral sirolimus; sclerotherapy*
8	M	7	Enlargement of the leg	Right leg	Microcystic LM	Oral sirolimus; open excision; endoscopic excision; sclerotherapy	Debulking	219	50	Elastic compression
9	F	10	Enlargement of thigh	Left thigh	Microcystic LM	Oral sirolimus	Debulking	209	200	Elastic compression
10	F	26	Pain from extensive superficial venous thrombosis; Achilles contracture	Right leg	Venous malformation	Open excision; sclerotherapy	Thrombectomy and gastrocnemius recession	201	100	Stretching exercises
11	F	18	Mass and pain; deformity of ankle dorsiflexion	Right leg	FAVA of extensor longus digitorum	Ethanol and bleomycin sclerotherapy	Radical resection and tendon transfer	213	100	None
12	M	10	Mass and pain; Achilles contracture	Left leg	FAVA of soleus	Partial excision	Radical resection	66	10	None
13	F	7	Mass	Right forearm	Venous malformation with thrombosis of flexor carpi ulnaris and flexor digitorum sublimes	Sclerotherapy	Radical resection	172	60	None
14	M	10	Mass and pain; knee contracture	Left thigh	FAVA of vastus medialis muscle	Ethanol and bleomycin sclerotherapy	Radical resection	213	5	Stretching exercises
15	F	8	Mass and pain; wrist contracture	Right forearm	FAVA of flexor carpi ulnaris	Ethanol and bleomycin sclerotherapy; oral sirolimus; partial excision	Thrombectomy; partial resection	104	20	Oral sirolimus; stretching exercises

Outcome

Fifteen patients underwent single-site endoscopic surgery for soft tissue lesions. Operative duration ranged from 66 to 455 minutes (median = 183 minutes). Blood loss ranged from 5 to 700 mL (median = 50 mL). One patient received a blood transfusion. The median follow-up duration after surgery was three months. In patients who had local pain from superficial venous thrombosis in KTS and venous malformation, thrombectomy was clinically curative. In most patients, the surgery was not cure. For patients with extensive lesions, severe morphologic changes of the limbs were commonly complicated; therefore, the surgery was debulking. All patients successfully underwent surgical debulking.

After thrombectomy, local edema and pain from superficial venous thrombosis disappeared during follow-up. With surgical debulking, the morphologic appearance of enlarged limbs was significantly improved. In patients who underwent muscular and tendinous surgeries, the normal or near-normal motion of the joint was obtained after postoperative stretching exercises. No local skin burn, incisional infection, or seroma occurred. Wound dehiscence was observed in one patient within 30 days after surgery.

## Discussion

Conventionally, surgery for soft tissue lesions is open excision. With the development of endoscopic concepts and techniques, more and more surgeries have become endoscopic. The natural lumen of the body, such as the gastrointestinal tract, urinary and genital tract, and airway, has been accessed by endoscopists and surgeons. The second space, such as the peritoneal cavity, thoracic cavity, brain ventricle, and articular cavity, has also been endoscopied by surgeons. More recently, endoscopists have accessed the third space, intramural or submucosal space [7,8]. The third space is not a natural cavity, it is created by an endoscopist. From the surgeon’s perspective, soft tissue space can also be created and studied by endoscopes [4]. The subcutaneous and intermuscular space is a broad potential surgical field that remains underexploited. Endoscopic surgery in this space can be considered soft tissue endoscopic surgery.

Trans-axillary endoscopic thyroidectomy and endoscopic breast surgery are typical endoscopic surgeries of soft tissue [9,10]. Other soft tissue endoscopic surgeries in the literature included endoscopic excision of dermoids [11-13], osteomas [13], and lipomas [2,13-15] using laparoscopic instruments, arthroscopic devices, or da Vinci surgical robots. We have developed endoscopic resection for vascular anomalies since 2016 [5]. Until now, various conditions of the soft tissue have been endoscopied in the literature [11,16-20]. As the endoscopic technique evolves at our center, we have developed single-site endoscopic surgery [21].

Single-site endoscopic surgery for soft tissue has some distinct features from multi-portal endoscopic surgery. Our recent study has demonstrated that the operative duration of endoscopic surgery was longer than that estimated for the excision of these lesions through a conventional open incision [21]. A significant portion of the surgery time is occupied by the creation of the subcutaneous space and the very large size of the lesion. In the step of creating space during single-site endoscopic surgery, dividing and partial resection of the lesion via the small incision can be considered as conventional open surgery. Therefore, the operative duration may be shortened. On the other hand, some multi-portal endoscopic surgeries could be called stealth surgeries, since portal incisions were made away from the lesion [3,12,15,22]. However, the incision for single-site endoscopic surgery was just made on the overlying skin of the lesion. So, single-site endoscopic surgery is less stealthy in some terms.

Single-site endoscopic surgery for soft tissue can be considered as endoscopy-assisted small incision surgery to some extent. If circumferential visualization and dissection of the lesion are presumed endoscopically difficult, the incision can be made on the overlying skin of this part. The difficult part of the lesion can be then open divided or exposed via the incision. Compared with multi-portal endoscopic surgery, more complicated surgeries can be accomplished through a single-site endoscopic approach.

Some studies demonstrated that soft tissue endoscopic surgery offers the advantages of promoting faster habilitation and improved patient satisfaction, improving cosmetic outcomes, and reducing postoperative wound healing time [21,22]. Among multi-portal, single-site endoscopic surgery and open surgery, we did not compare operative duration and complications, since different conditions were indicated for these approaches at our center. Patients who have undergone open surgery had more complicated conditions or more severe diseases. In a recent study, the incidence of wound complications in the open surgery group was slightly higher than that in the endoscopic surgery [21]. In a retrospective study, there was no significant difference in the total complication rate between the two groups [22].

We believe our study opens a new window of application of endoscopic surgery to various soft tissue diseases. This endoscopic surgery concept may provide valuable references for clinicians of other specialties, such as general surgery, pediatric surgery, vascular surgery, plastic surgery, dermatologic surgery, and orthopedics, as it can be used for resecting other subcutaneous and muscular lesions and reconstructing function of some joints. However, follow-up duration in this review is limited; therefore, symptom control and disease recurrence require constant vigilance.

## Conclusions

This retrospective review demonstrates the feasibility of using a single-site endoscopic approach to resection of subcutaneous, muscular and tendinous lesions and proof of principle for future soft tissue surgery.
